# Direct healthcare resource utilisation, health-related quality of life, and work productivity in patients with moderate rheumatoid arthritis: an observational study

**DOI:** 10.1186/s12891-021-04110-1

**Published:** 2021-03-13

**Authors:** James Galloway, Julie Edwards, Shweta Bhagat, Ben Parker, Ai Lyn Tan, James Maxwell, Mike Wallington, Sophee Blanthorn-Hazell, Claire Bellamy, Zoe Cole

**Affiliations:** 1 Centre for Rheumatic Diseases, King’s College London, London, UK; 2 Research and Development Department, Royal Wolverhampton NHS Trust, Wolverhampton, UK; 3 West Suffolk NHS Foundation Trust, Bury Saint Edmunds, UK; 4 Kellgren Centre for Rheumatology, NIHR Manchester Biomedical Research Centre, Manchester University Hospitals NHS Foundation Trust, Manchester Academic Health Science Centre, Manchester, UK; 5 NIHR Leeds Biomedical Research Centre, Chapel Allerton Hospital, Leeds Teaching Hospitals NHS Trust, Leeds, UK; 6 Sheffield Teaching Hospitals, Sheffield, UK; 7 OPEN VIE, Marlow, UK; 8 AbbVie Limited, Maidenhead, UK; 9 Rheumatology Department, Salisbury District Hospital, Salisbury, UK

**Keywords:** Rheumatoid arthritis, Quality of life, EQ-5D, Burden, Moderate, DAS28, Patient-reported outcome, Resource utilisation, Caregiver

## Abstract

**Background:**

The aim was to describe the population of patients with moderate rheumatoid arthritis (RA) in the United Kingdom and the burden of disease from the perspectives of the patient, caregiver, and health service.

**Methods:**

In this descriptive study, retrospective patient-level data were extracted from hospital medical records to assess healthcare resource utilisation and validated outcome measures were administered via questionnaire to patients with moderate RA (Disease Activity Score [DAS28] between 3.2 and 5.1) from eight secondary care centres, and their caregivers. Patient-reported outcome instruments were scored according to licensed manuals.

**Results:**

Outcome measures were completed by 102 patients and 38 caregivers. The mean EuroQoL-5 dimension-5 level crosswalk index value for patients was 0.62 (SD 0.24) compared to an England population norm of 0.82. Mean pain VAS score was 37.7 (SD 24.0) and mean Health Assessment Questionnaire Disability Index was 1.1 (SD 0.8). In employed patients who completed the Work Productivity and Activity Impairment questionnaire (*n* = 26), a mean 29% (SD 26%) reduction in work productivity was recorded. Patients experienced significant fatigue as a result of their RA (median Functional Assessment of Chronic Illness Therapy fatigue score 17.2 of a possible 52, interquartile range [IQR] 11.0–28.8). Over 50% of caregivers reported providing > 7 h of support care per week to the patient with RA, and 16 and 11% took paid/unpaid leave or reduced working hours, respectively. Mean Caregiver Reaction Assessment subscale scores were 1.9 (SD 0.9) for finance, 1.7 (SD 0.8) for health, 2.3 (SD 1.0) for schedule disruption, and 1.9 (SD 0.8) for family support. Patients had a mean 5.5 (SD 4.1) outpatient attendances and a median 9.0 (IQR 2.0–20.0) diagnostic and monitoring tests in the 12 months prior to enrolment.

**Conclusions:**

This study shows that moderate RA has a considerable impact on healthcare resources and on patients’ and caregivers’ lives. There is scope to improve the management of patients with moderate RA.

**Supplementary Information:**

The online version contains supplementary material available at 10.1186/s12891-021-04110-1.

## Background

Rheumatoid arthritis (RA) is an autoimmune disorder that affects more than 400,000 adults (approximately 1%) in the United Kingdom (UK) [1]. Chronic inflammation results in pain, stiffness, and swelling in the joints, which over time can lead to cartilage damage and joint destruction [2–4].

RA is associated with physical and psychological disabilities and poor quality of life and poses a substantial burden on patients and healthcare resources [5, 6]. Depression occurs in 13–42% of patients with RA [7], while the prevalence of fatigue in RA ranges from 40 to 90% [8, 9]. RA has a significant impact on work productivity with important socio-economic consequences [10, 11]. One study showed that 36–84% of individuals with RA take sickness absence due to their condition, and up to 50% stop work altogether over a period of 4.5–22 years despite wanting to remain in employment [12]. In the UK, 10% of early RA patients left work over a median of 3 years of follow-up [13]. Increasing disease severity has been associated with worsening disability, pain, fatigue, quality of life, and work and activity impairment [14]. The total (direct and indirect) cost of RA to the UK economy has been estimated at between £3.8 and £4.8 billion (US$5.1–6.4 billion) [1, 2], while the annual burden of RA across Europe is estimated to be approximately €3–5000 per patient (US$3589-5982) in non-drug costs [15].

The National Institute for Health and Care Excellence (NICE) recommends first-line treatment with conventional disease-modifying antirheumatic drug (cDMARD) monotherapy using oral methotrexate, leflunomide, or sulfasalazine. Additional cDMARDs are recommended as combination therapy when the treatment target (remission or low disease activity [LDA]) has not been achieved despite dose escalation. Short-term bridging treatment with glucocorticoids should be considered when a new cDMARD is started [16]. Biologic (bDMARDs) and targeted synthetic DMARDs are only recommended in the UK following an inadequate response to a combination of cDMARDs in patients with severe RA (Disease Activity Score [DAS28] > 5.1) [16]. This recommendation is more restrictive than European guidelines, which advise starting bDMARDs after a first cDMARD strategy has failed [17]. The strict prescription and reimbursement rules in the UK results in low bDMARD use, which is lower than countries with comparable Gross Domestic Product per capita (France, Japan) [18].

The European League Against Rheumatism (EULAR), as well as NICE, endorse the ‘treat-to-target’ principle for RA and recommends that the primary treatment goal in RA should be clinical remission (i.e. absence of signs and symptoms of significant inflammatory disease activity) or LDA in order to maximise long-term health-related quality of life (HRQoL) [16, 17]. Studies have demonstrated that patients with moderate RA on bDMARDs are more likely to achieve remission or LDA than patients with severe RA [19], leading to significant cost savings in terms of medical visits, laboratory tests, drug costs, hospitalisation, and days lost from work [20–22]. However, this is difficult to achieve in UK patients with an inadequate repsonse to cDMARDs and a DAS28 < 5.1, as access to bDMARDs is restricted.

There is a paucity of evidence from the UK on burden of disease in patients who are not eligible for advanced therapies based on current access restrictions (hereafter referred to as those with moderate RA disease activity). This information is important to help understand unmet needs in this population and to guide future clinical management. Thus, we aimed to describe this population of patients with moderate RA in the UK and the burden of disease from the perspectives of the patient, caregiver, and health service.

## Methods

### Study design

A multi-centre study was conducted at eight secondary care centres in the UK, including seven in England and one in Scotland. The study comprised a series of cross-sectional patient-reported outcome (PRO) questionnaires and matched caregiver questionnaires and a retrospective review of patient-level data from hospital medical records.

### Patient and caregiver recruitment

Eligible patients were identified either prospectively (selected consecutively at a routine clinic visit) or retrospectively (via the participating site’s clinical database, in reverse chronological order based on the date of their most recent DAS28 score) by members of the direct care team between June 2018 and March 2019. Patients who met the following criteria were included in the study: ≥18 years of age with a DAS28 score between 3.2 and 5.1 at the time of study enrolment (or at the most recent recording up to 8 weeks prior to enrolment), confirmed diagnosis of RA at least 2 years prior to enrolment, and who received a cDMARD in the 24 months prior to enrolment. For the purposes of this study, moderate RA was defined as a DAS28 > 3.2 ≤ 5.1. The exclusion criteria included DAS28 score > 5.1 recorded at any point during the 12 months prior to enrolment, previous biologic therapy or Janus kinase inhibitor for any condition, current or previous participation (≤5 years) in an interventional clinical trial for RA, patient under the care of the rheumatology department at the participating centre for less than 12 months prior to enrolment, and/or unable or unwilling to give consent for study participation. A sample size of 100 patients was deemed sufficient to enable the estimation of endpoints with adequate precision and also pragmatic based on the number of eligible patients expected for the study to give a representative sample of patients with moderate RA.

Caregivers were included if they were aged ≥18 years, met the definition of an informal caregiver (a spouse, adult child, other relative, partner, neighbour or friend who has a personal relationship with and provides a broad range of unpaid assistance to an adult with RA) at the time of study enrolment, and were the patient’s primary caregiver (defined as the person who provides the most unpaid assistance to the patient for their RA). Caregivers themselves unable or unwilling to give consent for completion of study questionnaires or those who cared for patients unable or unwilling to give consent were excluded from the study.

### Data collection

Data extracted from hospital medical records included patient demographics and clinical characteristics, disease severity, comorbidities, disease and treatment history, and data on healthcare resource utilisation (HCRU) for a minimum of 12 months and up to 24 months prior to study enrolment (where data were available). The date of RA diagnosis and number of cDMARDs received may have preceded this time period but were still captured in this study even if the date of diagnosis was more than 24 months prior to enrolment. Source data verification (SDV) was performed on a 15% random sample of patients across all participating centres to ensure data accuracy.

Questionnaires were either administered to the patient at their routine clinic visit or by post. Validated questionnaires comprised the following: 5-dimensional EuroQoL (EQ-5D-5L), Health Assessment Questionnaire Disability Index (HAQ-DI), Pain Visual Analogue Scale (VAS), Functional Assessment of Chronic Illness Therapy - Fatigue (FACIT-F), Morning Joint Stiffness Scale (MJS), and Work Productivity and Activity Impairment (WPAI) questionnaire.

These questionnaires have been shown to have excellent psychometric properties, including validity and reliability, for use in RA [23–27].

The EQ-5D-5L measures HRQoL (index score ranges from worse than dead [< 0] to full health [1]) and comprises the following five dimensions: mobility, self-care, usual activities, pain/ discomfort, and anxiety/depression [28]. In addition, a VAS is used to assess patient’s self-rated health status from ‘the worst health you can imagine’ (score 0) to ‘the best health you can imagine’ (score 100) with a one-day recall period. The HAQ-DI was used to assess functional ability and is composed of 20 items belonging to eight categories (dressing and grooming, getting up, eating, walking, hygiene, reach, grip, and daily activities) [29]. Scores of 0 to 1 are generally considered to represent mild to moderate disability, 1 to 2 represent moderate to severe disability, and 2 to 3 represent severe to very severe disability. The Pain VAS (0 indicates ‘no pain’ and 100 indicates ‘worst pain imaginable’) was used to measure the level of pain in the previous 24 h [30]. The 40-item FACIT-F comprises five subscales including physical (score range 0–28), social/family (0–28), emotional (0–24), and functional well-being (0–28), and the symptom-specific subscale for fatigue (0–52) [31, 32]. The total FACIT-F score ranges from 0 to 160, and higher scores represent better well-being for all scales/subscales. The MJS scale comprises two single-item measures that assess the length of time (in hours and minutes) and severity (0 indicates ‘no joint stiffness’ and 10 indicates ‘joint stiffness as bad as you can imagine’) of morning joint stiffness that patients experienced on the day of questionnaire completion [23]. The WPAI comprises six sections which are combined into four domains, including absenteeism, presenteeism, work productivity loss, and activity impairment [33]. Higher percentages show increased impairment. In addition to the validated questionnaires, patients were also asked about any change in employment status as a result of their RA and activities of daily living they require support with.

Caregivers were also asked to complete the EQ-5D-5L to assess the impact of providing care on caregiver HRQoL and the caregiver-specific WPAI to assess the impact looking after the RA patient had on caregiver work productivity. In addition, the validated Caregiver Reaction Assessment (CRA), which comprises five subscales (impact on schedule, health, finances, caregiver esteem, and family support), was used to assess the impact of caring on the caregiver [34]. The total and five subscale scores range from 1 to 5, where higher scores indicate more substantial impact of providing support. Caregivers were also asked about the number of hours of support provided to the patient, the activities of daily living that the patient requires support with, and changes in caregiver employment as a result of providing care to the patient with RA. A Patient Advisory Group reviewed the patient and caregiver questionnaires.

### Statistical analyses

Statistical analyses were performed using Stata version 14.2 (StataCorp, College Station, TX). Initial data processing tasks (e.g. quality checks and validations) and tabulation of results were conducted in Microsoft Excel. All analyses were performed with the available data with missing data reported. Quantitative variables approximating a normal distribution and variables describing hospital resource utilisation are presented as mean (standard deviation [SD]); other quantitative variables are presented as medians (interquartile range [IQR]). Categorical variables are described with frequencies and percentages. Distributions are presented as appropriate to the variable reported. The cost of HCRU was calculated using National Health Service (NHS) national schedule of reference costs (2017/18) [35]. The lowest cost of each drug referenced in the British National Formulary was used for analysis of medication costs [36]. Validated PRO instruments were scored according to licensed manuals, and missing data were handled in accordance with developer’s recommendations.

## Results

### Study population

**Fig. 1:**
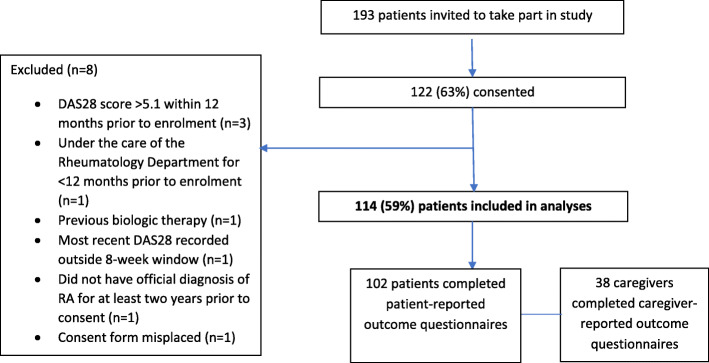
Flow chart of patient selection

A total of 114 patients were included in the analyses. A flow diagram of patient selection is presented in Fig. 1. The mean age of included patients was 55.5 (SD 13.9) years and 75% (85/114) were female (Table 1). The median disease duration was 7.8 (IQR 3.8–13.6) years at study enrolment (*n* = 108) and 5.1 (IQR 2.3–10.9) years at current cDMARD initiation (*n* = 102). All patients had a DAS28 score between 3.2 and 5.1 at their visit closest to enrolment (mean 3.8 [SD 0.5]) indicating moderate disease activity at the time of recruitment. Only 10% (7/70) and 17% (12/70) of DAS28 scores in the 12 months prior to enrolment indicated remission or LDA, respectively; 73% (51/70) indicated moderate disease. Patients had more tender joints (median 3.0 [IQR 2.0 to 5.0]) than swollen joints (median 1.0 [IQR 1.0 to 3.0]). Patients had a median of 2.0 (IQR 1.0 to 2.0) comorbidities at enrolment; 15% (*n* = 17) had no comorbidities. The most common comorbidity was cardiovascular disease (*n* = 33, 29%), followed by respiratory disease (*n* = 28, 25%), osteoporosis (*n* = 13, 11%), depression (*n* = 7, 6%), and diabetes (*n* = 6, 5%). Half of patients received two cDMARDs (mean 2.1 [SD 0.8]) between RA diagnosis and the start of the observation period (24 months prior to enrolment). The mean number of cDMARDs received between RA diagnosis and enrolment was 2.4 (SD 0.9).

**Table 1:** Patient demographics and clinical characteristics (*n* = 114)

Demographic/clinical variables	Number of patients (%)^**a**^
Age (years) at diagnosis	*N* = 108^b^
Mean (SD)	55.5 (13.9)
Gender	
Female	85 (75)
DAS28 score closest to enrolment	
< 2.6	0 (0)
2.6 < 3.2	0 (0)
3.2 ≤ 5.1	114 (100)
> 5.1	0 (0)
Mean (SD)	3.8 (0.5)
Number of swollen joints closest to enrolment	*N* = 111
0–1	57 (51)
2–4	43 (39)
≥5	11 (10)
Median (IQR)	1.0 (1.0–3.0)
Number of tender joints closest to enrolment	*N* = 111
0–1	21 (19)
2–4	56 (50)
≥5	34 (31)
Median (IQR)	3.0 (2.0–5.0)
Number of comorbidities at enrolment	
0	17 (15)
1	33 (29)
2	39 (34)
3	11 (10)
4	11 (10)
≥ 5	3 (3)
Median (IQR)	2.0 (1.0–2.0)
Number of cDMARDs received between RA diagnosis and start of observation period^c^	
None recorded	1 (1)
1	25 (22)
2	56 (49)
3	28 (25)
4	3 (3)
≥ 5	1 (1)
Mean (SD)	2.1 (0.8)

*cDMARDs* Conventional disease-modifying anti-rheumatic drugs, *DAS* Disease activity score, *IQR* Interquartile range, *RA* Rheumatoid arthritis, *SD* Standard deviation

^a^Results reported as n (%) unless otherwise indicated (e.g. mean, median)

^b^Six patients did not have a date of diagnosis recorded

^c^Start of observation period is 24 months prior to enrolment

### Patient-reported outcome measures

Of 114 patients included in the study, 102 (89%) completed PRO questionnaires. The PRO results are presented in Table 2. All PROs show some level of impairment in patients with moderate RA. The mean EQ-5D-5L crosswalk index value for patients with moderate RA was 0.62 (SD 0.24), lower than the population norm (0.82, age 55–64 in England). The mean EQ-5D-5L VAS score in this cohort was 62.2 (SD 20.0).

**Table 2:** Patient-reported outcomes among patients diagnosed with moderate RA (*n* = 102)

Patient-reported outcome measure	Value
EQ-5D-5L, mean (SD)	
EQ-5D crosswalk index value (*n* = 102)	0.62 (0.24)
VAS (*n* = 99)	62.2 (20.0)
WPAI, median (IQR)	
Absenteeism^a^ (*n* = 26)	0 (0–1)
Presenteeism^b^ (*n* = 28)	20 (10–40)
Work productivity loss^c^ (*n* = 26)	20 (10–48)
Activity impairment (*n* = 91)	40 (10–60)
Pain VAS (*n* = 99), mean (SD)	37.7 (24.0)
FACIT-F, median (IQR)	
Overall (0–160; *n* = 94)	97 (83.6–104.6)
Physical well-being sub-score (0–28; *n* = 102)	21 (14.0–24.0)
Social/family well-being sub-score (0–28; *n* = 95)	23 (17.5–26.6)
Emotional well-being sub-score (0–24; *n* = 99)	18 (14.0–20.0)
Functional well-being sub-score (0–28; *n* = 100)	18 (12.0–23.0)
Fatigue subscale (0–52; *n* = 98)	17.2 (11.0–28.8)
Morning Joint Stiffness	
Duration (hours; *n* = 94), median (IQR)	1.0 (0.3–2.0)
Severity (*n* = 101), median (IQR)	4.0 (2.0–6.0)
HAQ-DI, mean (SD)	1.1 (0.8)
Activities of daily living requiring support, n (%)	
Gripping/opening things	65 (64)
Housework	48 (47)
Reaching for/picking up things	46 (45)
Shopping	44 (43)
Gardening	39 (38)
Cooking and preparing food	37 (36)
Attending healthcare appointments	37 (36)
Getting up from sitting or lying down	27 (26)
Dressing/grooming	23 (23)
Moving around outdoors	22 (22)
Taking medication	15 (15)
Moving around indoors, including stairs	14 (14)
Washing and hygiene	13 (13)
Leisure activities	13 (13)
Eating/drinking	8 (8)
Other	5 (5)
Employment status change (in patients who were employed prior to RA diagnosis, *n* = 54) because of moderate RA^d^, n (%)	
Stopped work	14 (26)
Reduced work hours	11 (20)
Took paid leave	2 (4)
Took unpaid leave	1 (2)
Other	6 (11)
No changes in employment status because of RA^e^	22 (41)

*EQ-5D-5L* EuroQoL- 5 dimension, *FACIT-F* Functional Assessment of Chronic Illness Therapy - fatigue scale, *HAQ-DI* Health Assessment Questionnaire Disability Index, *IQR* Interquartile range, *RA* Rheumatoid arthritis, *SD* Standard deviation, *VAS* Visual analogue scale, *WPAI* Work Productivity and Activity Impairment

^a^% work time missed

^b^% work time lost due to reduced effectiveness

^c^Absenteeism + presenteeism

^d^Not mutually exclusive

^d^Includes patients who were already retired

**Fig. 2:**
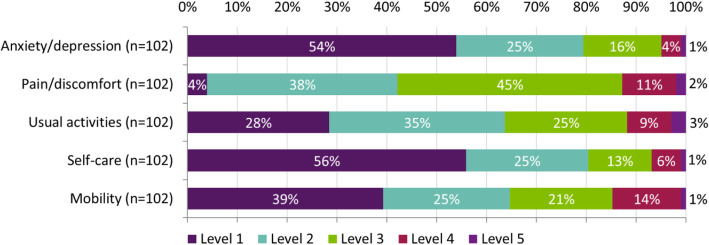
Patients’ quality of life (EQ-5D-5L) by domain and level. *Level 1 = no problems; level 2 = slight problems; level 3 = moderate problem; level 4 = severe problems; level 5 = extreme problems

**Fig. 3:**
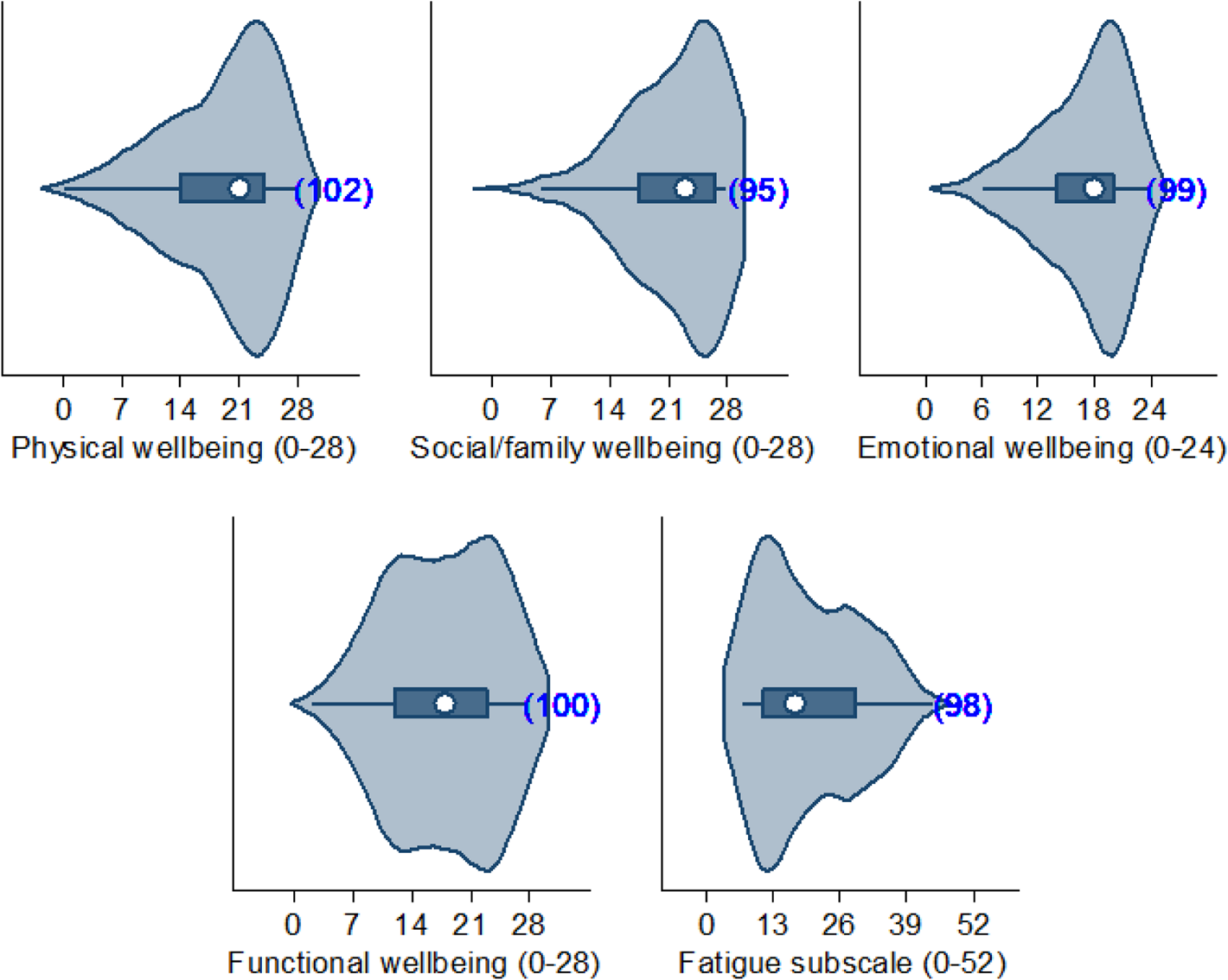
Violin plots showing FACIT-F subscale scores. The median, interquartile range, range, and kernel density are displayed, and the blue numbers in parentheses represent the number of responses

Most patients reported some level of impairment (i.e. levels 2–5) for the EQ-5D domains of pain/discomfort (98/102, 96% of patients), usual activities (73/102, 72%), and mobility (61%; Fig. 2). Patients reported a mean pain VAS score of 37.7 (SD 24.0) indicating moderate pain, and mean HAQ-DI of 1.1 (SD 0.8) showing moderate disability. Thirty-four changes in employment due to RA were recorded since diagnosis in 54 (59%) patients who were employed prior to RA diagnosis; 14 stopped work and 11 reduced their working hours. Fourty-1 % (*n* = 22) of patients reported no change in employment status due to moderate RA. In those who were still employed and completed the WPAI (*n* = 26), a mean 29% (SD 26%) reduction in work productivity was recorded. The median FACIT-F overall score for patients with moderate RA was 97.0 (IQR 83.6–104.6). The violin plots presented in Fig. 3 show that patients experienced significant fatigue as a result of their RA (median 17.2 [IQR 11.0–28.8]). The median duration of MJS was 1.0 (IQR 0.3–2.0) hour, and the median MJS severity score was 4.0 (IQR 2.0–6.0) indicating moderate stiffness. Activities of daily living that patients most commonly required assistance with included gripping or opening things (64%), housework (47%), reaching for/picking up things (45%), and shopping (43%).

### Caregiver-reported outcome measures

Thirty-eight caregivers completed questionnaires (see Table e1 in Additional file 1); mean age was 65.1 (SD 12.7) years and 65% (24/67) were male. The mean EQ-5D-5L crosswalk index value for caregivers was 0.80 (SD 0.16), and mean EQ-5D-5L VAS score was 76.3 (SD 16.9). Most caregivers reported no problems for most of the EQ-5D domains, including self-care (*n* = 36, 95%), usual activities (*n* = 28, 74%), anxiety/depression (*n* = 26, 68%), and mobility (*n* = 26, 68%); however, 71% (*n* = 27) reported slight or moderate pain or discomfort. Median scores for caregivers across the WPAI domains were 0% except for activity impairment which was 10% (IQR 0–45%). Mean CRA subscale scores for finance (1.9 [SD 0.9]), health (1.7 [SD 0.8]), schedule disruption (2.3 [SD 1.0]), and family support (1.9 [SD 0.8]) indicate mild to moderate negative impact on the caregiver. Fifty-six percent (20/36) of caregivers provided > 7 h of support care per week to the patient with RA, while 8% (3/36) provided > 35 h of care per week. Sixty-one percent of caregivers (11/18) reported no change in their employment status as a result of caring for the person with moderate RA; however, five caregivers (28%) took paid and/or unpaid leave and three (17%) stopped work or reduced working hours.

### Healthcare resource utilisation in 12 months prior to enrolment

Patients had a mean of 5.5 (SD 4.1) outpatient attendances in the 12 months prior to enrolment (Table 3); routine review was the most common reason (301/627, 48%). The healthcare professionals most commonly seen during outpatient attendances in the 12 months prior to enrolment were phlebotomists (313/822, 38% of attendances), specialist nurses (182/822, 22%), and consultants (144/822, 18%). The mean number of diagnostic and monitoring tests per patient was 13 (SD 13.3). There was only one inpatient admission and no A&E attendances.

**Table 3:** Direct healthcare resource utilisation in 12 months prior to enrolment (*n* = 114)

	Number of patients (%)
Number of outpatient attendances	
1 < 3	33 (29)
3 < 6	39 (34)
6 < 9	15 (13)
9 < 12	15 (13)
12 < 15	8 (7)
≥ 15	4 (4)
Mean (SD)	5.5 (4.1)
Number of telephone contacts	
0	86 (75)
1 < 3	23 (20)
3 < 6	2 (2)
6 < 9	3 (3)
≥ 9	0 (0)
Mean (SD)	0.5 (1.3)
Number of day case admissions	
0	107 (94)
1	6 (5)
2	1 (1)
Mean (SD)	0.1 (0.3)
Number of inpatient admissions	
0	113 (99)
1	1 (1)
Number of A&E attendances	
0	114 (100)
Number of diagnostic and monitoring tests^a^	
0	16 (14)
1 < 10	43 (38)
10 < 20	25 (22)
20 < 30	18 (16)
30 < 40	7 (6)
≥ 40	5 (4)
Mean (SD)	13.0 (13.3)

*A&E* Accident & emergency, *IQR* Interquartile range, *SD* Standard deviation

^a^Includes full blood count, C-reactive protein, liver function, renal function, erythrocyte sedimentation rate, X-ray, and others

All patients received treatment with ≥1 cDMARD in the 12 months prior to enrolment (mean 1.8 [SD 0.7]). Fifty-four percent (61/114) of patients had no new cDMARD initiated or dose change to ongoing cDMARD in the 12 months prior to enrolment. Patients had a mean of 3.0 (SD 1.3) RA treatments (including cDMARDs and other RA treatments) in the 12 months prior to enrolment. Ongoing treatments at enrolment included cDMARDS such as methotrexate (*n* = 93, 82%), hydroxychloroquine (*n* = 48, 42%), sulfasalazine (*n* = 31, 27%), leflunomide (*n* = 9, 8%), and azathioprine (*n* = 1, 1%), and other RA treatments including corticosteroids (*n* = 7, 6%), other painkillers (*n* = 43, 38%), nonsteroidal anti-inflammatory drugs (NSAIDs; *n* = 25, 22%), and other (*n* = 5, 4%). The total median HCRU cost per patient was £567 (IQR £361–1031) during the 12-month observation period. The highest median HCRU cost per patient in the 12-month period prior to enrolment was for outpatient attendances, costing £336 (IQR £243–516) per patient (see Table e2 in Additional file 2).

## Discussion

This real-world study examines the burden of moderate RA in UK patients who are not eligible for advanced therapies based on current access restrictions. Our results suggest that moderate RA in these patients has a considerable impact on the utilisation of healthcare resources and on patients’ and caregivers’ lives, which approaches that of patients with severely active RA.

Patient demographics were generally consistent with those reported from the national, prospective, ongoing British Society of Rheumatology Biologics Register for Rheumatoid Arthritis; however, patients were slightly younger than patients with RA registered in the UK Biobank cohort [37, 38]. The median disease duration was shorter than that of other UK studies reported in the literature [37, 39]. Comorbidities were similar to those reported for patients with RA by the UK Biobank (e.g. depression, diabetes); however, a higher proportion of patients had cardiovascular comorbidities in this study [38]. This likely reflects a patient sampling and data recoding issue; however, cardiovascular risk is an important comorbidity in RA and has historically been attributed to disease activity thus this is an area that warrants further study.

Whilst patients were not excluded from the study if they had a DAS28 score indicating mild RA in the 12 months prior to enrolment, only 10 and 17% of DAS28 scores in the last 12 months were in remission or LDA, respectively. The remaining 73% were moderate which implies most patients had persistently moderate disease.

### Patient- and caregiver-reported outcomes

Patients with moderate RA showed some level of impairment in all PRO measures used in our study. Despite treatment, many patients reported significantly impaired HRQoL, as demonstrated by the EQ-5D-5L mean index score of 0.62 compared to an England population norm of 0.82. This is consistent with a previous report by Pavelka et al. which reported a mean EQ-5D-5L index score of 0.59 despite patients having on average more severe disease than that seen in our study (mean DAS28 score 4.4 versus 3.8 in our study) [40]. The EQ-5D-5L we report is higher than that reported in a Swedish (EQ-5D-5L mean index score 0.34; mean DAS28 5.0) and a wider European study (EQ-5D-5L mean index score 0.53) [41, 42]. The HRQoL is affected by the level of severity as measured by DAS28 and other factors including culture, socioeconomic status, perception, healthcare system, and social structure. The VAS of 62.2 was consistent with previous reports in 54 patients with RA (46% with high and 41% with moderate disease activity) [43]. Pain (96%) and inability to do usual activities (73%) were key drivers for HRQoL impairment according to EQ-5D in our study.

The FACIT fatigue subscale score for this study (17.2) was lower than those reported by Pavelka et al., Smolen et al. (mean DAS28 4.4), and Žagar et al. (32.4, 33.7, and 28.7, respectively) indicating that patients in this study experienced significantly more fatigue as a result of their RA despite a lower mean DAS28 [40, 43, 44]. Corticosteroids are a driver of fatigue; however, only 6% of patients had ongoing treatment with corticosteroids at enrolment. Hence, it is unlikely the burden of fatigue observed in this study was due to steroid treatment. The presence of comorbidities or the contribution of undiagnosed low mood are alternative explanations. The possibility that this is a chance finding due to small sample size cannot be ruled out.

Patients in our study experienced moderate disability (mean HAQ-DI score 1.1). Mean HAQ-DIs of 1.2, 1.1, 1.2, and 1.0 were recorded in other studies with higher mean DAS28 scores of 4.4, 4.4, 5.0, and 4.5, respectively [23, 40, 44, 45]. The median duration (1.0 h) and moderate severity of morning joint stiffness in our patients was also comparable to other studies where the mean DAS28 was slightly higher at 4.5 and 4.4 [23, 44]. Together, these data suggest that the burden of disease in this cohort approaches that of patients with severely active RA. This study demonstrates the burden of disease and exposes an opportunity to improve lives. Recognising the patient impacts of moderate disease allows clinicians to appreciate the potential benefits that could be achieved if remission was attained [18]. A study which assessed the impact of biologics on RA disease activity and quality of life showed that Kuwaitis who had easy access to biologics reported significantly better treatment outcomes including lower numbers of swollen joints and DAS28 scores compared to non-Kuwaitis [46].

Total work productivity loss (mean 29% in this study versus 33.5 and 43.2%) and activity impairment (mean 37% versus 48.1 and 56.2%) were lower than that reported in other studies which included patients with more severe disease [42, 47]. However, work productivity loss was higher than that reported in a Latin American study (mean 8.6% across Argentina, Brazil, Colombia, and Mexico) despite most patients classified as having high severity [48]. A European cross-sectional study of the impact of disease severity and duration on cost, early retirement, and ability to work in RA reported greater levels of work impairment with increased disease activity and pain level [5]. An assessment of the association between work impairment and other variables would be interesting but this was beyond the scope of our study. We show that moderate RA still has a significant impact on patients’ ability to work with WPAI values comparable to other chronic illnesses, such as chronic obstructive pulmonary disease, asthma and irritable bowel syndrome [49]. Almost half of patients in our cohort had changes in employment due to their RA and in those who remained employed and completed the WPAI, a mean 29% reduction in work productivity was recorded. This has high cost implications as it has recently been suggested that the greatest impact on costs for patients with RA is reduced performance while working (at-work productivity loss) [50].

Data from this study also show a significant impact on caregivers looking after patients with moderate RA. Over 50% of caregivers provided > 7 h of support care per week to the patient with RA, mean CRA subscale scores indicate mild to moderate negative impact on the caregiver, and 16 and 11% took paid and/or unpaid leave or reduced working hours, respectively. There are little data available on caregiver burden in RA. Here we show that the burden of moderate RA extends beyond the patient.

### Healthcare resource utilisation

Data on HCRU for patients with moderate RA are sparse within the literature. Existing data show similar levels of day case admissions (mean 0.2 in the literature versus 0.1 in the 12 months prior to enrolment in our study) and cDMARD utilisation (mean 1.43 in the literature versus 1.8 in our study) [39]. It should be noted however, that in the period reviewed in this study the ‘treat-to-target’ principle may not have been fully implemented. The distribution of cDMARDs is consistent with previous UK data [39]. The median disease duration was 5.1 years at current cDMARD initiation, and over half of patients had not had a dose change to their cDMARD therapy in the last year, despite having moderate disease. This combined with the fact that patients received a mean of 2.4 cDMARDs between RA diagnosis and enrolment may imply a lack of additional effective options for these patients. Relatively frequent outpatient attendances and diagnostic/monitoring tests in the 12-month period prior to enrolment, and the fact that all patients were receiving treatment with cDMARDs in this period suggests that the management of moderate RA places a significant burden on the NHS.

### Strengths and limitations

This study represents one of the most comprehensive attempts to measure burden of disease in a population of UK patients with moderate RA. We used a range of validated PRO measures and also considered caregiver and health service perspectives to give an all-round better picture of the burden of moderate RA in the UK. Some limitations however also need consideration. First, the sample may not be representative. The consent rate was 63%, and the characteristics of the patients who consented may not be representative of the wider population of patients with moderate RA. Patients included in this study were under the care of physicians and study sites that were willing to participate and therefore these centres may not be representative of current wider UK secondary care clinical practice. Another possible limitation is that the interpretation of data collected retrospectively is dependent on the completeness and quality of the medical records and the reliability of data abstraction. We employed SDV in this study to identify and correct abstraction errors. Furthermore, patient-reported data are subject to recall bias; however, this likely had little impact on the results as only short recall was required. Finally, due to only 6% of patients having ongoing treatment with corticosteroids at enrolment, the results may not be generalisable to other non-UK cohorts where steroid use is more prevalent.

## Conclusions

In conclusion, we show that moderate RA requires frequent hospital visits for clinic appointments and has a considerable impact on the lives of patients (and their caregivers) who are not eligible for advanced therapies based on current access restrictions. The impact (for fatigue, disability, and pain/discomfort) is comparable to that seen in patients on the more severe end of the RA disease spectrum. This suggests that there is substantial opportunity for patients within this disease severity spectrum. The information presented here will aid decision making around the cost benefit of different therapeutic strategies for this often overlooked RA subpopulation.

## Supplementary Information

**Additional file 1: Supplementary Table e1.** on “Caregiver-reported outcomes amongst caregivers looking after patients with moderate RA” provided in Word .doc format.

**Additional file 2: Supplementary Table e2.** on “Costs for healthcare resource use in the 12 months prior to enrolment” provided in Word .doc format.

## Data Availability

As patient consent for data sharing was not obtained in this study we are unable to provide access to the data. Upon reasonable request an anonymised form of the data may be made available from the corresponding author.

## Abbreviations

A&E: Accident and Emergency
bDMARDs: Biologic disease-modifying antirheumatic drugs
CRA: Caregiver Reaction Assessment
cDMARDs: Conventional disease-modifying antirheumatic drugs
DAS28: Disease Activity Score
EQ-5D-5L: EuroQoL-5 dimension-5 level
EULAR: The European League Against Rheumatism
FACIT-F: Functional Assessment of Chronic Illness Therapy - fatigue scale
HAQ-DI: Health Assessment Questionnaire Disability Index
HCRU: Healthcare resource utilisation
HRQoL: Health-related quality of life
IQR: Interquartile range
LDA: Low disease activity
MJS: Morning Joint Stiffness Scale
NHS: National Health Service
NICE: National Institute for Health and Care Excellence
NSAIDs: Nonsteroidal anti-inflammatory drugs
PRO: Patient-reported outcome
RA: Rheumatoid arthritis
SD: Standard deviation
SDV: Source data verification
UK: United Kingdom
VAS: Visual analogue scale
WPAI: Work Productivity and Activity Impairment questionnaire

## References

[1] Arthritis Research UK. State of musculoskeletal health 2017: Arthritis and other musculoskeletal conditions in numbers. Available from: http://www.arthritisresearchuk.org/arthritis-information/data-and-statistics/state-of-musculoskeletal-health.aspx. [cited 2018 Jan 8]

[2] National Institute for Health and Care Excellence. Rheumatoid arthritis in adults: management (CG79) . 2009. Available from: https://www.nice.org.uk/guidance/cg79 [cited 2018 Jan 8] 30102507

[3] Scott DL, Shipley M, Dawson A, Edwards S, Symmons DP, Woolf AD (1998). The clinical management of rheumatoid arthritis and osteoarthritis: strategies for improving clinical effectiveness. Br J Rheumatol.

[4] Rheumatoid arthritis - NICE CKS. Available from: https://cks.nice.org.uk/rheumatoid-arthritis#!backgroundsub:1. [cited 2018 Jan 17]

[5] Galloway J, Capron J-P, De Leonardis F, Fakhouri W, Rose A, Kouris I (2020). The impact of disease severity and duration on cost, early retirement and ability to work in rheumatoid arthritis in Europe: an economic modelling study. Rheumatol Adv Pract.

[6] Mandal M, Dasgupta A, Dutt D, Taraphdar P, Ghosh P, Paul B (2020). Quantification of health-related quality of life among patients with rheumatoid arthritis: an institution-based study in Kolkata, West Bengal. J Fam Med Prim Care.

[7] Margaretten M, Barton J, Julian L, Katz P, Trupin L, Tonner C (2011). Socioeconomic determinants of disability and depression in patients with rheumatoid arthritis. Arthritis Care Res.

[8] van Hoogmoed D, Fransen J, Bleijenberg G, van Riel P (2010). Physical and psychosocial correlates of severe fatigue in rheumatoid arthritis. Rheumatol Oxf Engl.

[9] Rupp I, Boshuizen HC, Jacobi CE, Dinant HJ, van den Bos GAM (2004). Impact of fatigue on health-related quality of life in rheumatoid arthritis. Arthritis Rheum.

[10] Li X, Gignac MAM, Anis AH (2006). The indirect costs of arthritis resulting from unemployment, reduced performance, and occupational changes while at work. Med Care.

[11] Gwinnutt JM, Leggett S, Lunt M, Barton A, Hyrich KL, Walker-Bone K (2020). Predictors of presenteeism, absenteeism and job loss in patients commencing methotrexate or biologic therapy for rheumatoid arthritis. Rheumatol Oxf Engl.

[12] Burton W, Morrison A, Maclean R, Ruderman E (2006). Systematic review of studies of productivity loss due to rheumatoid arthritis. Occup Med Oxf Engl.

[13] McWilliams DF, Varughese S, Young A, Kiely PD, Walsh DA (2014). Work disability and state benefit claims in early rheumatoid arthritis: the ERAN cohort. Rheumatol Oxf Engl.

[14] da Rocha Castelar Pinheiro G, Khandker RK, Sato R, Rose A, Piercy J (2013). Impact of rheumatoid arthritis on quality of life, work productivity and resource utilisation: an observational, cross-sectional study in Brazil. Clin Exp Rheumatol.

[15] National Rheumatoid Arthritis Society and University of Chester. The Burden of Rheumatoid Arthritis across Europe: a Socioeconomic Survey (BRASS) - Summary Report. Available from: https://www.nras.org.uk/data/files/Publications/Surveys%20Reports/UoC_HCD_BRASS%20Summary%20Report%20FINAL.pdf. [cited 2018 Feb 6]

[16] Rheumatoid arthritis in adults: management | Guidance and guidelines | NICE. Available from: https://www.nice.org.uk/guidance/ng100. [cited 2019 Feb 18].

[17] Smolen JS, Breedveld FC, Burmester GR, Bykerk V, Dougados M, Emery P (2016). Treating rheumatoid arthritis to target: 2014 update of the recommendations of an international task force. Ann Rheum Dis.

[18] Bergstra SA, Branco JC, Vega-Morales D, Salomon-Escoto K, Govind N, Allaart CF (2018). Inequity in access to bDMARD care and how it influences disease outcomes across countries worldwide: results from the METEOR-registry. Ann Rheum Dis.

[19] Kavanaugh A, Keystone E, Greenberg JD, Reed GW, Griffith JM, Friedman AW (2017). Benefit of biologics initiation in moderate versus severe rheumatoid arthritis: evidence from a United States registry. Rheumatol Oxf Engl.

[20] Hallert E, Husberg M, Skogh T (2011). 28-joint count disease activity score at 3 months after diagnosis of early rheumatoid arthritis is strongly associated with direct and indirect costs over the following 4 years: the Swedish TIRA project. Rheumatol Oxf Engl.

[21] Beresniak A, Gossec L, Goupille P, Saraux A, Bamberger M, Bregman B (2011). Direct cost-modeling of rheumatoid arthritis according to disease activity categories in France. J Rheumatol.

[22] Barnabe C, Thanh NX, Ohinmaa A, Homik J, Barr SG, Martin L (2013). Healthcare service utilisation costs are reduced when rheumatoid arthritis patients achieve sustained remission. Ann Rheum Dis.

[23] Bacci ED, DeLozier AM, Lin C-Y, Gaich CL, Rooney T, Hoffman R (2017). Psychometric properties of morning joint stiffness duration and severity measures in patients with moderately to severely active rheumatoid arthritis. Health Qual Life Outcomes.

[24] Zhang W, Bansback N, Boonen A, Young A, Singh A, Anis AH (2010). Validity of the work productivity and activity impairment questionnaire--general health version in patients with rheumatoid arthritis. Arthritis Res Ther.

[25] Husni M, Kosinski M, Rendas-Baum R, Kafka S, Han C, Chan E, et al. Psychometric Properities of the patient related outcome measure FACIT-fatigue in rheumatic arthritis and psoriatic arthritis: a literature review. Arthritis Rheumatol. 2019;71(suppl 10). Available from: https://acrabstracts.org/abstract/psychometric-properities-of-the-patient-related-outcome-measure-facit-fatigue-in-rheumatic-arthritis-and-psoriatic-arthritis-a-literature-review/.

[26] Fries JF, Spitz P, Kraines RG, Holman HR (1980). Measurement of patient outcome in arthritis. Arthritis Rheum.

[27] Renskers L, van Uden RJJC, Huis AMP, Rongen SAA, Teerenstra S, van Riel PLCM (2018). Comparison of the construct validity and reproducibility of four different types of patient-reported outcome measures (PROMs) in patients with rheumatoid arthritis. Clin Rheumatol.

[28] Herdman M, Gudex C, Lloyd A, Janssen M, Kind P, Parkin D (2011). Development and preliminary testing of the new five-level version of EQ-5D (EQ-5D-5L). Qual Life Res Int J Qual Life Asp Treat Care Rehab.

[29] Linde L, Sørensen J, Ostergaard M, Hørslev-Petersen K, Hetland ML (2008). Health-related quality of life: validity, reliability, and responsiveness of SF-36, 15D, EQ-5D [corrected] RAQoL, and HAQ in patients with rheumatoid arthritis. J Rheumatol.

[30] Hawker GA, Mian S, Kendzerska T, French M (2011). Measures of adult pain: visual analog scale for pain (VAS pain), numeric rating scale for pain (NRS pain), McGill pain questionnaire (MPQ), short-form McGill pain questionnaire (SF-MPQ), chronic pain grade scale (CPGS), short Form-36 bodily pain scale (SF-36 BPS), and measure of intermittent and constant osteoarthritis pain (ICOAP). Arthritis Care Res.

[31] Cella D, Lai J-S, Chang C-H, Peterman A, Slavin M (2002). Fatigue in cancer patients compared with fatigue in the general United States population. Cancer..

[32] Cella D, Yount S, Sorensen M, Chartash E, Sengupta N, Grober J (2005). Validation of the functional assessment of chronic illness therapy fatigue scale relative to other instrumentation in patients with rheumatoid arthritis. J Rheumatol.

[33] WPAI scoring, Reilly Associates. Available from: http://www.reillyassociates.net/WPAI_Scoring.html. [cited 2019 Apr 17]

[34] Given CW, Given B, Stommel M, Collins C, King S, Franklin S (1992). The caregiver reaction assessment (CRA) for caregivers to persons with chronic physical and mental impairments. Res Nurs Health.

[35] Reference costs | NHS Improvement. Available from: https://improvement.nhs.uk/resources/reference-costs/. [cited 2019 Mar 22]

[36] BNF: British National Formulary - NICE. Available from: https://bnf.nice.org.uk/. [cited 2019 Apr 17]

[37] Kearsley-Fleet L, Davies R, De Cock D, Watson KD, Lunt M, Buch MH (2018). Biologic refractory disease in rheumatoid arthritis: results from the British Society for Rheumatology biologics register for rheumatoid arthritis. Ann Rheum Dis.

[38] Cook MJ, Bellou E, Bowes J, Sergeant JC, O’Neill TW, Barton A (2018). The prevalence of co-morbidities and their impact on physical activity in people with inflammatory rheumatic diseases compared with the general population: results from the UK biobank. Rheumatol Oxf Engl.

[39] Watts RA, Mooney J, Barton G, MacGregor AJ, Shepstone L, Irvine L (2015). The outcome and cost-effectiveness of nurse-led care in the community for people with rheumatoid arthritis: a non-randomised pragmatic study. BMJ Open.

[40] Pavelka K, Szekanecz Z, Damjanov N, Majdan M, Nasonov E, Mazurov V (2013). Induction of response with etanercept-methotrexate therapy in patients with moderately active rheumatoid arthritis in central and Eastern Europe in the PRESERVE study. Clin Rheumatol.

[41] Jorgensen T, Turesson C, Kapetanovic M, Englund M, Turkiewicz A, Christensen R (2017). EQ-5D utility, response and drug survival in rheumatoid arthritis patients on biologic monotherapy: a prospective observational study of patients registered in the south Swedish SSATG registry. PLoS One.

[42] Taylor PC, Alten R, Gomez-Reino JJ, Caporali R, Bertin P, Sullivan E (2018). Clinical characteristics and patient-reported outcomes in patients with inadequately controlled rheumatoid arthritis despite ongoing treatment. RMD Open.

[43] Žagar I, Delimar V, Pap M, Perić D, Laktašić Žerjavić N, Perić P (2018). Prevalence and correlation of depressive symptoms with functional scores, therapy and disease activity among Croatian patients with rheumatoid arthritis: a preliminary study. Psychiatr Danub.

[44] Smolen JS, Strand V, Koenig AS, Szumski A, Kotak S, Jones TV (2016). Discordance between patient and physician assessments of global disease activity in rheumatoid arthritis and association with work productivity. Arthritis Res Ther.

[45] Twigg S, Hensor EMA, Emery P, Tennant A, Morgan AW (2017). Yorkshire early arthritis register consortium. Patient-reported outcomes as predictors of change in disease activity and disability in early rheumatoid arthritis: results from the Yorkshire early arthritis register. J Rheumatol.

[46] Al-Herz A, Saleh K, Al-Awadhi A, Al-Kandari W, Hasan E, Ghanem A, et al. Accessibility to biologics and its impact on disease activity and quality of life in patients with rheumatoid arthritis in Kuwait. Clin Rheumatol. 2020. 10.1007/s10067-020-05444-2. 10.1007/s10067-020-05444-2 PMC8102280 33044725

[47] Hone D, Cheng A, Watson C, Huang B, Bitman B, Huang X (2013). Impact of etanercept on work and activity impairment in employed moderate to severe rheumatoid arthritis patients in the United States. Arthritis Care Res (Hoboken).

[48] Xavier RM, Zerbini CAF, Pollak DF, Morales-Torres JLA, Chalem P, Restrepo JFM (2019). Burden of rheumatoid arthritis on patients’ work productivity and quality of life. Adv Rheumatol Lond Engl.

[49] Miller PSJ, Hill H, Andersson FL (2016). Nocturia work productivity and activity impairment compared with other common chronic diseases. PharmacoEconomics..

[50] van Vilsteren M, Boot C, Knol D, van Schaardenburg D, Voskuyl A, Steenbeek R (2015). Productivity at work and quality of life in patients with rheumatoid arthritis. BMC Musculoskelet Disord.

