# Supplementary material for: Direct healthcare resource utilisation, health-related quality of life, and work productivity in patients with moderate rheumatoid arthritis: an observational study
Source: BMC Musculoskelet Disord. 2021 Mar 13;22:277. doi: 10.1186/s12891-021-04110-1 (PMC7956119; doi:10.1186/s12891-021-04110-1)
Supplement: Supplementary file 1 — Additional file 1: Supplementary Table e1. on “Caregiver-reported outcomes amongst caregivers looking after patients with moderate RA” provided in Word .doc format. [file 12891_2021_4110_MOESM1_ESM.docx]

**Table e1. Caregiver-reported outcomes amongst caregivers looking after patients with moderate RA (n=38)**

| **Caregiver-reported outcome measure** | **Value** |
| --- | --- |
| EQ-5D-5L, mean (SD) |  |
| EQ-5D crosswalk index value | 0.80 (0.16) |
| VAS | 76.3 (16.9) |
| WPAI, median (IQR) |  |
| Absenteeism (n=11) | 0% (0-0) |
| Presenteeism (n=12) | 0% (0-8) |
| Work productivity loss (n=11) | 0% (0-10) |
| Activity impairment (n=37) | 10% (0-45) |
| CRA, mean (SD) |  |
| CRA total score | 2.4 (0.5) |
| Self-esteem | 4.0 (0.6) |
| Impact on finances | 1.9 (0.9) |
| Impact on health | 1.7 (0.8) |
| Disrupted schedule | 2.3 (1.0) |
| Lack of family support | 1.9 (0.8) |
| Activities of daily living requiring support (n=37), n (%) | N=37 |
| Gripping/opening things | 31 (84) |
| Housework | 27 (73) |
| Reaching for/picking up things | 24 (65) |
| Shopping | 25 (68) |
| Gardening | 24 (65) |
| Cooking and preparing food | 22 (59) |
| Attending healthcare appointments | 22 (59) |
| Getting up from sitting or lying down | 15 (41) |
| Dressing/grooming | 14 (38) |
| Moving around outdoors | 9 (24) |
| Taking medication | 12 (32) |
| Moving around indoors, including stairs | 7 (19) |
| Washing and hygiene | 8 (22) |
| Leisure activities | 6 (16) |
| Eating/drinking | 4 (11) |
| Other | 5 (14) |
| Number of hours of support per week (n=36), n (%) | N=36 |
| Fewer than 7 hours per week | 16 (44) |
| 7 to 14 hours per week | 9 (25) |
| 15 to 24 hours per week | 5 (14) |
| 25 to 34 hours per week | 3 (8) |
| More than 35 hours per week | 3 (8) |
| Employment status change because of moderate RA^*^, n (%) |  |
| Stopped work | 3 (8) |
| Reduced work hours | 1 (3) |
| Took paid leave | 2 (5) |
| Took unpaid leave | 4 (11) |
| Other | 8 (21) |
| No changes in employment status because of RA | 23 (61) |

*^*^Not mutually exclusive; CRA = Caregiver Reaction Assessment; EQ-5D-5L = EuroQoL- 5 dimension; IQR = Interquartile range; RA = Rheumatoid arthritis; SD = Standard deviation; VAS = Visual analogue scale; WPAI = Work Productivity and Activity Impairment*
