# Supplementary material for: Direct healthcare resource utilisation, health-related quality of life, and work productivity in patients with moderate rheumatoid arthritis: an observational study
Source: BMC Musculoskelet Disord. 2021 Mar 13;22:277. doi: 10.1186/s12891-021-04110-1 (PMC7956119; doi:10.1186/s12891-021-04110-1)
Supplement: Supplementary file 2 — Additional file 2: Supplementary Table e2. on “Costs for healthcare resource use in the 12 months prior to enrolment” provided in Word .doc format. [file 12891_2021_4110_MOESM2_ESM.docx]

Table e2. Costs for healthcare resource use in the 12 months prior to enrolment

| **Costs (per patient) associated with HCRU** | **Outpatient  attendances*** | | **Telephone  contacts** | | **Day case  attendances** | | **Inpatient admissions**  **(elective)** | | **RA therapies** | | **Total cost per patient** | |
| --- | --- | --- | --- | --- | --- | --- | --- | --- | --- | --- | --- | --- |
|  | **n** | **% (N=114)** | **n** | **% (N=114)** | **n** | **% (N=114)** | **n** | **% (N=114)** | **n** | **% (N=114)** | **n** | **% (N=114)** |
| No HCRU recorded | 0 | 0% | 86 | 75% | 107 | 94% | 113 | 99% | 2 | 2% | 0 | 0% |
| <£200 | 18 | 16% | 23 | 20% | 7 | 6% | 0 | 0% | 71 | 62% | 4 | 4% |
| £200<£400 | 50 | 44% | 2 | 2% | 0 | 0% | 0 | 0% | 14 | 12% | 35 | 31% |
| £400<£600 | 31 | 27% | 2 | 2% | 0 | 0% | 0 | 0% | 5 | 4% | 21 | 18% |
| £600<£800 | 7 | 6% | 1 | 1% | 0 | 0% | 0 | 0% | 11 | 10% | 13 | 11% |
| £800<£1,000 | 2 | 2% | 0 | 0% | 0 | 0% | 0 | 0% | 10 | 9% | 9 | 8% |
| £1,000<£1,200 | 4 | 4% | 0 | 0% | 0 | 0% | 0 | 0% | 1 | 1% | 31 | 27% |
| ≥£1,200 | 2 | 2% | 0 | 0% | 0 | 0% | 1 | 1% | 0 | 0% | 1 | 1% |
| **Total** | **114** |  | **114** |  | **114** |  | **114** |  | **114** |  | **114** |  |
| Mean | £406 | | £38 | | £7 | | £18 | | £260 | | £728 | |
| SD | £263 | | £97 | | £28 | | £193 | | £305 | | £512 | |
| Median | £336 | | £0 | | £0 | | £0 | | £107 | | £567 | |
| IQR | £243 to £516 | | £0 to £19 | | £0 to £0 | | £0 to £0 | | £42 to £380 | | £361 to £1,031 | |
| Range | £96 to £1,675 | | £0 to £610 | | £0 to £192 | | £0 to £2,065 | | £0 to £1,192 | | £110 to £3,908 | |
| **Total cost (12 months): £83,036** | | | | | | | | | | | | |

**Outpatient attendances include cost of tests and procedures where applicable*

*HCRU = Healthcare resource utilisation; IQR = Interquartile range; RA = Rheumatoid arthritis; SD = Standard deviation*
